# Advances on Bioanalysis: Recent Approaches in the Determination of Biomarkers, Drugs of Abuse and Medicines

**DOI:** 10.3390/molecules27103188

**Published:** 2022-05-17

**Authors:** Mário Barroso, Eugenia Gallardo, Luís A. Passarinha

**Affiliations:** 1 Serviço de Química e Toxicologia Forenses, Instituto de Medicina Legal e Ciências Forenses-Delegação do Sul, 1169-201 Lisboa, Portugal; 2 Centro de Investigação em Ciências da Saúde, Universidade da Beira Interior (CICS-UBI), Av. Infante D. Henrique, 6201-556 Covilhã, Portugal; 3 Laboratório de Fármaco-Toxicologia, Ubimedical, Universidade da Beira Interior, Estrada Municipal 506, 6200-284 Covilhã, Portugal; 4 UCIBIO-Applied Molecular Biosciences Unit, Departamento de Química, Faculdade de Ciências e Tecnologia, Universidade NOVA de Lisboa, 1099-085 Caparica, Portugal; 5 Associate Laboratory i4HB-Institute for Health and Bioeconomy, NOVA School of Science and Technology, Universidade NOVA, 2819-516 Caparica, Portugal

## 1. Introduction

New developments in instrumental approaches, for instance, hyphenated techniques, have allowed great advances in the bioanalytical field over the last half century, and there is no doubt that toxicology was one of the most improved areas. Indeed, synthetic chemicals have been increasing in both variety and volume at a more rapid dissemination, and the consumption of drugs of abuse is a growing phenomenon. The available data allow us to conclude that consumption patterns are becoming more complex, since consumers have facilitated access to a greater number of substances. This situation is potentially much more dangerous to human health due to the abuse of new and unknown substances and to the interaction between them and/or their effects.

Synthetic cannabinoids are a good example of this situation, as can be seen by the reported deaths in 2020 related to their consumption and the alerts issued by public health authorities on the matter, including the appearance of adulterated material with high potency compounds within the natural cannabis market [1]. Great efforts have been directed towards a better knowledge of these compounds’ toxicokinetics [2] and to assess their long-term deleterious effects. These compounds are only a small part of all the substances to which we are exposed and whose toxicity and toxicokinetic profiles are unknown. Advances in omics technologies have been widely used in life sciences and related fields, such as cell biology, neurobiology and others, such as toxicology, which has contributed to a better knowledge of the behavior and fate of toxic compounds in the body. Specifically, proteomics and metabolomics provide an outstanding approach to understand cell physiology under stress conditions and by identifying and comparing quantitatively target proteins in several biological matrices. Metabolomic approaches have been used in toxicology as well, showing that comparative metabolic profiles can play a crucial role in the research and validation of biomarkers. In addition, it may help in the understanding and interpretation of xenobiotics’ mechanisms of action. Moreover, proteomics offer complementary information to genomics and transcriptomics, and it is essential at the molecular level to study complex biochemical processes, facilitating recognizing structures and functions proteins and understand the mechanisms of damage.

These advances are not exclusive to analytical instrumentation, however. Indeed, different developments have been also related to sample preparation approached, aiming at isolating and separating analytes from the biological matrices where they are. Miniaturized techniques or different sampling approaches (e.g., dried matrix spots) have high interest taking into account the ease of sample manipulation, process speed and low cost per analysis due to the consumption of low amounts of organic solvents; this last feature is also important in terms of environment.

This Special Issue of Molecules, “Advances on Bioanalysis: Recent Approaches in the Determination of Biomarkers, Drugs of Abuse and Medicines”, aims at improving the knowledge within this scientific field, gathering a collection of papers focused on recent developments in the isolation and identification of biomarkers, drugs of abuse and medicines from biological specimens. New extracting approaches were used, and applications adding value to the areas of clinical diagnosis and toxicology have been proposed.

## 2. Contribution

This Special Issue gathers nine research papers and three reviews, covering developments in bioanalysis and sample preparation, detection and quantification of disease biomarkers and toxic agents and drug monitoring approaches with applications in different analytical fields.

Amante et al. [3] describe an untargeted metabolomics approach to study metabolic patterns potentially related to fentanyl exposure. HepG2 cell lines were incubated with either fentanyl or common drugs of abuse, and a cohort of 96 samples was created. These samples were subjected to untargeted analysis by ultra-high-performance liquid chromatography combined with time-of-flight mass spectrometry; 81 additional urine samples, including negative controls and positive samples obtained from recent drug users, were studied as well. The authors concluded that the possible identification of diagnostic indirect biomarkers may possibly lead to the development of more sensitive and specific class-targeted methods in the future. One limitation of the study was the lack of urine samples from subjects consuming fentanyl several days before sample collection, when the parent drug and its direct metabolites would no longer be detectable in urine. Still in the area of metabolomics, Krokos et al. [4] have evaluated the cytotoxicity of cocaine by means of metabolomics-based analyses in HepG2 cells. The authors monitored about 106 metabolites from different metabolic pathways. Multivariate analysis revealed potential biomarkers in the extracellular and intracellular samples. A predominant effect of cocaine administration on the metabolic pathways of alanine, aspartate and glutamate was observed. The authors have succeeded in revealing metabolic changes following cocaine administration using targeted metabolomics; however, deciphering the exact cytotoxic mechanism of cocaine still remains a challenge. Cao et al. [5] have studied myocardial injury induced by chronic alcohol consumption and the underlying mechanism of alcoholic cardiomyopathy using approaches based on histopathology, echocardiography, molecular biology and metabolomics. By means of metabolomic analysis of myocardium specimens, 297 differentially expressed metabolites were identified, and these were involved in KEGG pathways related to the biosynthesis of unsaturated fatty acids, vitamin digestion and absorption, oxidative phosphorylation, pentose phosphate and purine and pyrimidine metabolism. This study demonstrated that chronic alcohol consumption disordered cardiomyocyte structure, caused thinning and dilation of the left ventricle and decreased cardiac function. The study further provides an insight into the causes of myocardial injury due to chronic alcohol exposure.

With application in the diagnosis of diseases, He et al. [6] established a new ^1^H NMR-based method to quantify trimethylamine-*N*-oxide (TMAO), a gut-derived metabolite that has been found to be associated with enhanced risk for atherosclerosis and cardiovascular disease, and betaine in serum and food samples. The key step included sample preparation using a selective solid-phase extraction (SPE) column for the retention of basic metabolites. Briefly, the research team performed a 1D-NOESY pulse sequence to suppress solvent signal to enhance resonance sensitivity of metabolites. Thereon, NMR sensitivity and resolution were further enhanced by using an SPE procedure before sample analysis. Cation-exchange sorbent selectively absorbed alkaline metabolites, such as TMAO, betaine and choline, and successfully removed the interferences of glucose and taurine, signals of which were severely overlapped with those of the targets. Overall, the established method was validated with a good linearity, precision, repeatability, stability and accuracy. The limits of detection and quantification for TMAO and betaine were much lower than the levels usually present in normal human serum, suggesting the developed ^1^H NMR method had good application in clinical settings. In addition, this analytical method was successfully applied to detect the serum levels of TMAO and betaine in TMAO-fed mice and high-fructose-fed rats and was also used to determine the contents of TMAO and betaine in several kinds of food, such as fish, pork, milk and egg yolk. Overall, the SPE column effectively minimized peak overlap, and ^1^H NMR analysis has shown potential as a fast and reliable method to detect TMAO and betaine in several biological matrices.

These manuscripts are an excellent starting point to open the implementation of untargeted metabolomics in forensic toxicology, as well in the diagnosis of diseases.

Xia et al. [7] take advantage of proteomics histopathology and biochemical analysis to explore the role of oxidative stress in hepatotoxicity induced by nutmeg abuse. Nutmeg is a traditional spice and medicinal plant with a variety of pharmacological activities. In a previous metabolomics report, the authors proved the hepatotoxicity of nutmeg and demonstrated that a high dose of nutmeg could affect the synthesis and secretion of bile acids and could cause oxidative stress. Here, the results showed and proved that a high dose (4 g/kg) of nutmeg can cause significant increased level of CYP450s and depletion of antioxidants, resulting in oxidative stress damage and lipid metabolism disorders. In addition, the increased level of malondialdehyde and decreased level of glutathione peroxidase were found after nutmeg exposure. The study demonstrated that exposure to high doses of nutmeg might cause lipid metabolism disorders and oxidative reactive stress, subsequent antioxidants depletion and, finally, hepatocyte damage. This process might be induced primarily by CYP450 in a dose-dependent fashion, and potential mechanisms for hepatotoxicity after nutmeg exposure were proposed. 

Brandon et al. [8] conducted an interesting study concerning the factors that influence the metabolism and pharmacokinetics of different synthetic cannabinoid receptor agonists. The authors have used both in silico and experimental methods to determine a number of parameters such as lipophilicity, short-term stability in plasma, plasma protein binding, *in vitro* intrinsic clearance, the structural and conformational features influencing their interaction with metabolic enzymes and their metabolic clearance rates. The authors have obtained *in vitro* pharmacokinetic data, which were used to estimate *in vivo* human hepatic clearance and hepatic extraction ratios, thus helping to predict their pharmacokinetics; this could be of high importance to toxicological casework.

Kunicki et al. [9] propose an approach to monitor mycophenolic acid in plasma using a simple and inexpensive high performance liquid chromatography coupled to an ultraviolet detector, technology easily available in hospital laboratories. The authors discuss the issue of whether internal standardization is strictly necessary for reliable quantitative analysis. Mycophenolic acid is an immunosuppressive agent that presents high inter- and intra-subject pharmacokinetic variability, and as such, its therapeutic monitoring may be of value in clinical practice. For sample preparation, the authors have used protein precipitation, and method validation followed the guidelines of the European Medicines Agency. The proposed method was considered an attractive alternative for both LC-MS/MS and immunochemical tests.

The assessment of greenness of analytical protocols assumes greater importance nowadays due to environmental issues, and this is observed in the development of new laboratorial approaches for routine application. Almalki et al. [10] present a study in which seven published chromatographic methods for the analysis of four neurotransmitters and their mixtures in several biological fluids and tissues are compared. The authors have used the National Environmental Method Index, Analytical Eco-Scale Assessment and Green Analytical Procedure Index for this comparation. 

Finally, a study on the stability of cocaine and opiates in oral fluid samples was presented by Almeida et al. [11]. The authors studied a number of parameters that could influence the stability of the target compounds, namely storage temperature, light, use of preservatives (and respective concentrations), and time. The effects of each parameter were evaluated using the design of experiments (DOE) approach. The authors concluded that, among the preservatives under study, sodium fluoride at 1% was the one that appeared to improve the stability of the analytes in oral fluid samples stored in the dried saliva spots (DSS). Additionally, the best conditions of storage were room temperature and with the presence of light (regular laboratory lamps). In addition, the authors propose a new sample preparation approach based on dried matrix spots and gas chromatography tandem mass spectrometry. The dried matrix spots sampling approach is acknowledged as a collection technique that simplifies transport and storage, since liquid matrices are dried on a filter paper.

As previously mentioned, this Special Issue also focused on the relevance of identifying and detecting traditional and emerging biomarkers for the diagnosis and monitoring of several pathologies. In this sense, Ornelas-González et al. [12] present a review on “Enzymatic Methods for Salivary Biomarkers Detection: Overview and Current Challenges”. Overall, this literature review shows a global vision of the biomarker pathway from the laboratory to the clinical applications, proposing enzymatic assays as a cost-effective alternative to overcome the limitations of current methods for the quantification of biomarkers in saliva, highlighting the considerations techniques and operations necessary for sampling, method development, optimization and validation. The authors provide a detailed description of the use of enzymatic methods for salivary biomarkers detection for the diagnosis of several pathologies, including cardiovascular diseases, Alzheimer’s disease, diabetes, oral diseases and cancer. In addition, they point to emerging technologies that will become routine in the clinic in the coming years, such as portable devices and tests for the non-invasive diagnosis of diseases, and also point the biosensors with optical, electrochemical and piezoelectric transducers for the detection of biomarkers in saliva in short periods of time and with high accuracy and comment on the appearance of new functional devices taking advantage of nanotechnology developments. Complementarily and still in the domains of new biomarkers, Tian et al. [13] contributed with a review entitled “Circular RNAs in Sudden Cardiac Death Related Diseases: Novel Biomarker for Clinical and Forensic Diagnosis”. These authors reinforce that novel diagnostic biomarkers are urgently required to identify patients with early-stage cardiovascular diseases (CVD) and to assist in the postmortem diagnosis of SCD cases without typical cardiac damage. They also showed that an increasing number of studies show that circular RNAs (circRNAs) have stable expressions in myocardial tissue, which makes them potential CVD biomarkers. This research team briefly introduced the biogenesis and functional characteristics of circular RNAs (circRNAs) and described the roles of circRNAs in multiple SCD-related diseases (including coronary artery disease (CAD), myocardial ischemia or infarction, arrhythmia, cardiomyopathy and myocarditis) and discussed the application prospects and challenges of circRNAs as a novel biomarker in the clinical and forensic diagnosis of SCD, based on pericardial and vitreous body fluids. Among other reasons, these findings demonstrate that circRNAs are enriched in exosomes, which can be collected by several biological fluids such as blood, urine, saliva, breast milk and semen. Moreover, because of the competition with mRNA in the processing mechanism, tissue and body-fluid samples with few mRNA expression changes may have significant alteration in corresponding circRNA levels, which excavates new biomarker opportunities. 

Finally, lateral flow immunoassays and nucleic acid lateral flow assays in a wide variety of early diagnostic and screening applications have been thoroughly analyzed by Qriouet et al. [14] in the review “Monoclonal Antibodies Application in Lateral Flow Immunochromatographic Assays for Drugs of Abuse Detection”. Here, the research team outlined the production process of antibodies against drugs of abuse (such as heroin, amphetamine, benzodiazepines, cannabis, etc.) to be used in lateral flow immunoassays as revelation or detection molecules, with a focus on components, principles, formats and mechanisms of reaction. Furthermore, the perspective on aptamer use for lateral flow assay development was also discussed as a possible alternative to antibodies in view of improving the limit of detection, sensitivity and specificity of these assays and opens up prospects with great socioeconomic impacts.

Overall, this Special Issue of Molecules brings together great contributions to the desired continued advance in the determination of biomarkers, drugs of abuse and medicines and the development of new applications for these compounds in the area of bioanalysis.
